# More Paths to PI3Kγ

**DOI:** 10.1371/journal.pbio.1001594

**Published:** 2013-06-25

**Authors:** Len Stephens, Phillip Hawkins

**Affiliations:** The Babraham Institute, Cambridge, United Kingdom

In the huge collection of molecules that underpin mammalian biology, only a small number stand out as targets for drug development. PI3Kγ is one such molecule that has received substantial investments to assess whether inhibitors can be developed as potential therapeutics. Although many studies have addressed the structure and regulation of PI3Kγ, our understanding of the enzyme is far from complete. A publication in *PLOS Biology* from the Wymann group provides new insights into this process by identifying an alternate route to PI3Kγ activation, and these results enable us to understand how PI3Kγ signaling is regulated in mast cells and is thus important in so many cell, tissue, and disease settings.

## Roles for PI3Kγ in Health and Disease

PI3Kγ is expressed strongly in a number of immune cells, including mast cells, neutrophils, and eosinophils. In these cell types, it sits at a key, early, near-receptor stage in pro-inflammatory, intracellular signaling pathways. This is, in part, why genetic loss or selective inhibition of PI3Kγ has little effect on normal mouse development and function but can suppress inflammation in a number of mouse models of disease, including rheumatoid arthritis [1], anaphylaxis [2], atherosclerosis [3], and glomerulonephritis [4]. Interestingly, however, PI3Kγ seems to perform a variety of important roles in other cell types/systems where it is often barely detectable—for example, in the heart, where it suppresses cAMP signaling and contractility (and hence may be a useful therapeutic target in certain types of cardiac failure) [5]–[7], and in fat metabolism, where its activity supports fat deposition [8]. Finally, there is evidence that PI3Kγ may support tumour growth and progression [9],[10].

## Class I PI3K Signaling

PI3Kγ (phosphoinositide 3-kinase) belongs to the class I PI3K family of signaling enzymes (along with PI3Ks α, β, and δ) that 3-phosphorylate the membrane phospholipid PtdIns(4,5)P_2_ to yield the signaling lipid PtdIns(3,4,5)P_3_. The class I PI3Ks can all be activated by cell surface receptors to drive accumulation of PtdIns(3,4,5)P_3_ in the inner leaflet of the plasma membrane. This acts as a signal enabling proteins capable of binding PtdIns(3,4,5)P_3_, typically via PH domains, to concentrate at the cytosolic interface of the plasma membrane. The classic example of a PH domain-containing PtdIns(3,4,5)P_3_ effector is PKB (Akt), but there are possibly as many as 60–70 in a mammalian genome and up to at least 20 can be expressed in the same cell. This family of PtdIns(3,4,5)P_3_ effectors transduce the lipid signals into many forms, including changes in protein kinase, Rac-family-GEF and Rho-family-GAP activity, and/or distribution.

## PI3Kγ—The Detail

PI3Kγ is made up of a p110γ catalytic subunit [11] combined with either a p84 (also called p87^PIKAP^) [12]–[14] or a p101 regulatory subunit [15]. Interestingly, the three proteins are well conserved in eukaryotes from humans to fish; p84 and p101 have even retained their neighbouring genetic location. PI3Kγ is thought to exist as a dimer *in vivo*, although this is only based on the lack of any evidence for the existence of free p110γ. It appears that despite immune cells like neutrophils, expressing p101 and p84, mast cells, the subject of the study reported by the Wymann group, only express p84 [16].

## The p101 Regulatory Subunit

Our current understanding of PI3Kγ activation centers on how the p101 subunit allows the complex to be substantially activated by G-protein βγ subunits (Gβγs) liberated from G proteins upon activation of GPCRs. This mechanism is thought to explain why PI3Kγ is typically (but not universally) activated by GPCRs. Frustratingly, we do not know the residues/domains in either p101 or p110γ involved in the interaction with Gβγs. It appears the interaction between p101 and p110γ is very tight and unlikely to have a significant on/off rate under physiological conditions. There is some evidence supporting direct interactions between Gβγs and both p101 and p110γ [15],[17].

## The p84 Regulatory Subunit

But the involvement of the p84 in PI3Kγ has been less clear. There is anecdotal evidence in the field that the interaction between p84 and p110γ is less tight than that between p101 and p110γ. Despite this, once bound, p84/p110γ complexes are able to survive gel-filtration chromatography or multiple cycles of washing with traditional detergent-containing lysis buffers through pull-down protocols without significant dissociation, suggesting that their association is not rapidly reversible. It is clear, however, that the p84/p110γ heterodimer is substantially less sensitive to Gβγs (at least to a limited subset of Gβγs that have been tested) than the p101/p110γ complex [14],[18].

## Regulation by Ras

P110γ contains a classical Ras-binding domain (RBD) that has been demonstrated to bind selectively to Ras-GTP. Ras-GTP can activate p110γ, p101/p110γ, and p84/p110γ *in vitro* or in transfected cells [19]. Furthermore, this interaction is important *in vivo*, as demonstrated by the effect of knocking-in a Ras-insensitive, but Gβγ-sensitive, allele of p110γ in mice (p110γ*^DASAA/DASAA^*) [20]. There are lines of evidence which suggest that Ras-GTP (and not Gβγs) regulates p84/p110γ complexes in transfected cells, while p101/p110γ complexes are only regulated by Gβγs [18]. This view suggests that p84/p110γ complexes may be less exclusively controlled by GPCRs but rather by any ligand capable of activating Ras, including receptor tyrosine kinase pathways.

## Mast Cells

Mast cells are tissue-resident immune cells that express high-affinity receptors for IgE (FcεRI). Mast cell FcεRI are effectively permanently bound with IgE. These FcεRI/IgE complexes are activated by ligands that are specific targets for the variable regions of IgEs, including allergens. Once activated, mast cells can release a huge range of inflammatory mediators, including histamine, LTB4, PAF, and PGD2. The intracellular signals generated by activated FcεRIs are primarily based on protein tyrosine kinase–mediated mechanisms that would naturally lead to activation of the class IA PI3Ks such as PI3Kδ [21],[22]; however, PI3Kγ has been shown to have an important role in mast cell activation [2]. The role for PI3Kγ is through an autocrine/paracrine mechanism, involving among other things released adenosine working back on A3 (A_3_-Ars) GPCRs, that synergizes with the primary effects of FcεRI activation.

## Insights into Mast Cell Regulation of PI3Kγ Activation

A recent study by Walser et al. in *PLOS Biology* began with several threads, a key one of which was the ability of thapsigargin (a drug that causes a very selective receptor-independent increase in cytosolic Ca^2+^ by slowly releasing intracellular ER Ca^2+^ stores into the cytosol and activating Ca^2+^ influx via the store-operated-Ca^2+^-entry (SOCE) route) to stimulate phosphorylation of PKB in a PI3Kγ-dependent manner. That result was very unexpected and a variety of controls suggested it was unlikely to be dependent on release of the mast cell paracrine/autocrine factor adenosine acting back on A3 GPCRs.

**Figure 1:**
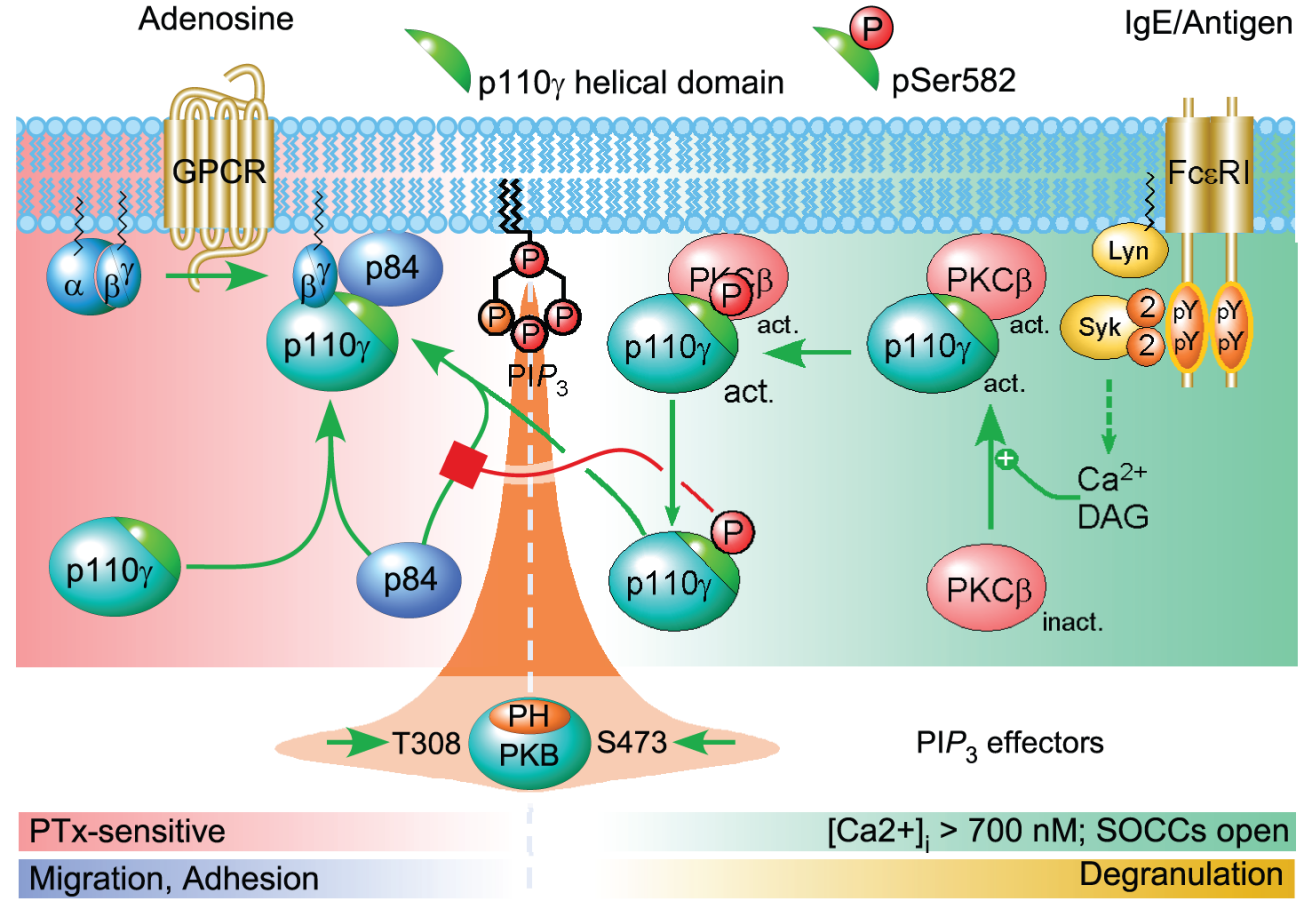
Mast cell environment regulates PI3Kγ activation. Coincident with migration and adhesion of mast cells, adaptor protein p84 relays the GPCR signal from GPCR-mediated dissociation of trimeric G proteins to activate the PI3Kγ complex. However, when mast cells degranulate, FcεRI receptors are clustered via IgE/antigen complexes, and a signaling cascade triggers intracellular Ca^2+^ store depletion and the opening of store-operated Ca^2+^ channels. The resulting increase in calcium ion concentration and PLCγ-derived diacylglycerol activates PKCβ, which binds to p110γ and subsequently phosphorylates Ser582. This phosphorylated p110γ can no longer interact with p84, and the PI3Kγ complex is therefore unresponsive to GPCR inputs. Image credit: Walser et al., 10.1371/journal.pbio.1001587

Wide-ranging experiments support a model (Figure 1) in which FcεRI cross-linking leads to a Ca^2+^ signal, involving SOCE, that drives activation of PKCβ. Through an interaction between PKCβ and the helical domain of p110γ, S582-p110γ is phosphorylated. This leads to both activation of the kinase activity of PI3Kγ (which leads to increased PIP_3_ and downstream signaling) and a reduction in the affinity of p110γ for p84. The outcome of these events is argued to be a rebalancing in the amount of GPCR-sensitive p84/p110γ and free, GPCR-insensitive p110γ, thus reducing the sensitivity of PI3Kγ to GPCRs. These conclusions represent major conceptual advances for the field. Beyond their major impact on our understanding of mast cell biology, they offer potential molecular explanations for the roles of PI3Kγ in a number of important, but ill-understood, physiological and disease settings. Furthermore, the paper introduces beautiful structural data from deuterium-exchange protection assays that reveal where the presence of p84 masks the surface of p110γ in its helical domain. Further biochemical experiments demonstrate the potential power of interactions with, or post translational modifications of, the helical domain of p110s to regulate class I PI3K function both in terms of changes in catalytic activity and binding of adaptor subunits. This type of mechanism may be a widely important mode of regulation in class I PI3K signaling.

As ever with first steps, many questions remain. The role of Ras in the activation of PI3Kγ in mast cells is unclear. Given that RasGrp1 (and RasGrp4) is relatively abundant in mast cells, and activated by a combination of PKC-mediated phoshorylation and diacylaglycerol (DAG) binding, and in lymphocytes is the primary mechanism driving activation of Ras downstream of antibody receptor activation [23],[24], it might have been expected to be involved in this pathway, but does not appear to be. This will be clarified by testing the impact of knocking-in a Ras-insensitive version of p110γ on mast cell responsiveness. The apparent contradiction between the simple model described above and the dogma that p84 is tightly bound to p110γ (that is supported by the data from the deuterium-exchange protection assays) needs to be resolved. It currently leaves it difficult to understand the pattern of events; does PKCβ need to compete with p84 to enable significant stoichiometries of phosphorylation to occur? As always, a high-resolution view of the p84/p110γ complex would be immensely useful.

## Concluding Remarks

The *PLOS Biology* paper from Walser et al. makes many important contributions. It reveals how intracellular signals from mast cell FcεRIs, classically thought to engage class IA PI3K signaling, can control p110γ directly via the PLC effectors Ca^2+^ and DAG/PKC. These effects are mediated by an interaction between PKCβ and the helical domain of p110γ that enables S582-p110γ to be phosphorylated and activate the lipid kinase activity of PI3Kγ. The outcome is the emergence of a new mechanism by which class I PI3Ks can be activated. The paper also provides a first insight into the interactions between p110γ and p84, hopefully the first step in the exploitation of deuterium-exchange methodologies to reveal more about the interactions of p101, p84, p110γ, and Gβγs. Finally, the work may offer molecular explanations for some of the many poorly understood roles that PI3Kγ fulfills outside of the immune system.
